# Controlling Injection Barriers for Ambipolar 2D Semiconductors via Quasi‐van der Waals Contacts

**DOI:** 10.1002/advs.201801841

**Published:** 2019-04-19

**Authors:** Junjun Wang, Feng Wang, Zhenxing Wang, Ruiqing Cheng, Lei Yin, Yao Wen, Yu Zhang, Ningning Li, Xueying Zhan, Xiangheng Xiao, Liping Feng, Jun He

**Affiliations:** ^1^ CAS Center for Excellence in Nanoscience CAS Key Laboratory of Nanosystem and Hierarchical Fabrication National Center for Nanoscience and Technology Beijing 100190 P. R. China; ^2^ University of Chinese Academy of Sciences Beijing 100049 P. R. China; ^3^ Key Laboratory of Semiconductor Materials Science Institute of Semiconductors Chinese Academy of Sciences No.A35, QingHua East Road Haidian District Beijing 100083 P. R. China; ^4^ Department of Physics Hubei Nuclear Solid Physics Key Laboratory Wuhan University Wuhan 430072 P. R. China; ^5^ State Key Lab Solidification Processing College of Materials Science and Engineering Northwestern Polytechnical University Xi'an Shaanxi 710072 P. R. China

**Keywords:** ambipolar 2D semiconductors, Fermi level pinning effect, Schottky barrier, substrate influence, van der Waals heterostructures

## Abstract

Barriers that charge carriers experience while injecting into channels play a crucial role on determining the device properties of van der Waals semiconductors (vdWS). Among various strategies to control these barriers, inserting a graphene layer underneath bulk metal may be a promising choice, which is still lacking experimental verification. Here, it is demonstrated that graphene/metal hybrid structures can form quasi‐van der Waals contacts (q‐vdWC) to ambipolar vdWS, combining the advantages of individual metal and graphene contacts together. A new analysis model is adopted to define the barriers and to extract the barrier heights in ambipolar vdWS. The devices with q‐vdWC show significantly reduced Schottky barrier heights and thermionic field emission activation energies, ability of screening the influence from substrate, and Fermi level unpinning effect. Furthermore, phototransistors with these special contacts exhibit enhanced performances. The proposed graphene/metal q‐vdWC may be an effective strategy to approach the Schottky–Mott limit for vdWS.

Van der Waals semiconductors (vdWS) are recognized as one of the most promising candidates for new‐generation electronic and optoelectronic devices because of the advantages of atomically thin thickness, dangling‐bond‐free surface, useful electrical and photoelectric properties, layer‐dependent tunable bandgap, and so forth.^1^, ^2^, ^3^, ^4^, ^5^ Considering the difficulties of controllable doping of the ultrathin bodies, vdWS with ambipolar conduction, like molybdenum telluride (MoTe_2_) and tungsten diselenide (WSe_2_), host a great potential to fabricate complementary logic devices on a single flake.^3^, ^6^, ^7^, ^8^ Nevertheless, the ambipolar properties of their devices always strongly depend on the metal contacts, substrates, and body thickness, showing only n‐terminal or p‐terminal dominant conduction behavior, losing the advantage.^9^, ^10^, ^11^

Schottky barrier (SB), an energy barrier that charge carriers have to overcome to inject into the channel from bulk metal, plays a crucial role in the electrical properties that vdWS devices show. Actually, it has been proved that ambipolar vdWS can be analytically captured by the so‐called Schottky barrier field effect transistor (SBFET) model.^12^, ^13^ Therefore, many efforts have been made to uncover the physical nature of vdWS/metal interface, and to realize high‐quality contacts with reduced Schottky barrier height (SBH).^14^, ^15^, ^16^ Approaching the limit of Schottky–Mott rule is the main strategy because of the absence of viable doping method. Even though the dangling‐bond‐free surface of vdWS, strong Fermi level pinning (FLP) effect caused by the metal induced gap states (MIGS) still exists due to the defects and crystal structure destruction of vdWS while depositing metal.^17^, ^18^ So far, a number of solutions have been proposed to eliminate the FLP effect. Replacing bulk metal with graphene (G) as contact electrode is a common method because of the ultrahigh carrier mobility and excellent mechanical properties of graphene,^19^, ^20^, ^21^ as well as strong work function tunability of vdWS/G system enabled by the tunable Fermi level of graphene^22^ and the van der Waals nature of the interface. However, small SBH can be only achieved while applying gate voltage, which limits its practical application.^23^ In a very recent work, predeposited metal thin films were transferred onto MoS_2_ flakes to eliminate the destructions caused by direct evaporation.^24^ Combined with using poly(methyl methacrylate) (PMMA) as dielectric environment, Schottky–Mott limit was approached on MoS_2_ by varying the types of contact metals.

Inserting van der Waals material buffer layer between bulk metal and 2D channel is another promising strategy, where van der Waals features could be kept at the channel/buffer layer interface.^25^, ^26^, ^27^ For instance, it has been shown that inserting h‐BN buffer layer enhances the electrons tunneling enabling high‐quality n‐type contacts to MoS_2_.^25^, ^27^, ^28^, ^29^, ^30^ But, owing to the insulating feature of h‐BN, the band alignment of the h‐BN/metal hybrid contacts cannot be tuned by electric field and solely defined by the metal used. On the other hand, using graphene as the buffer layer may be a better choice due to the possibility of combining the advantages of the van der Waals interface and strong tunability of graphene itself, which has recently been predicted by theoretical calculations.^29^, ^31^ Experimentally, Ni‐etched graphene^32^ and thick graphite^33^ have been separately used as buffer layer of electrode to study the contact resistance of MoS_2_ field effect transistor (FET). However, for the former, only special metal like Ni that has strong interaction with graphene can be adopted and an additional annealing processing is required to create zigzag‐terminated edges, which strongly limit its generalization. For the latter, the thick graphite (≈20 layers) might have eliminated the function of metal, leading to the result that the metal/graphite contacts are equivalent to the individual graphene contacts.^20^, ^34^ Therefore, more experimental works are urgently needed to fully reveal the properties of graphene/metal hybrid structure as contacts and to verify the prediction raised by theoretical calculations.

Here, we demonstrate that graphene/metal (G/M) hybrid structure can work as quasi‐van der Waals contacts (q‐vdWC) which combine the advantages of individual metal and graphene van der Waals contacts together. With this distinguishing feature, q‐vdWC shows the following advantages compared with two other structures: i) significantly reduced SBH and thermionic field emission activation energy to ambipolar vdWS regardless of the metal and substrate types, ii) immunity to the influence of substrate, and iii) Fermi level unpinning effect. Furthermore, the MoTe_2_ and WSe_2_ phototransistors with this special contacts exhibit very high responsivity up to 1012 and 1.33 × 10^4^ A W^−1^, respectively, which are among the highest performances of pure MoTe_2_ and WSe_2_ photodetectors.^34^, ^35^, ^36^, ^37^, ^38^, ^39^, ^40^ This work demonstrates that G/M quasi‐van der Waals contacts may be an effective solution to approach the Schottky–Mott limit.^24^

Devices used in this work are composed with mono‐ or few‐layer ambipolar vdWS and graphene stripes. Degenerately doped Si with 300 nm SiO_2_ substrates were used as global gates. For a direct comparison, three types of contacts were fabricated on a same device: individual metal (M), individual graphene (G), and graphene/metal (G/M) hybrid contacts. Here, G/M hybrid contacts were achieved through transferring G stripe arrays followed by evaporation of metals (see Experimental Section for details). It is worth noting that using preprepared G patterns is essential to avoid direct plasma etching on vdWS channels that would inevitably cause unpredictable damage and greatly degrade its electrical performance.^41^, ^42^ **Figure** **1**a depicts the schematic of our devices, where the green, gray, and yellow stripes represent vdWS channels, G, and metals, respectively. The inset shows the atomic structure view of the vdWS/G/M cross‐section. Remarkably, the up‐most deposited metals will metalize the G interlayer while the vdWS/G interface keeps van der Waals feature,^29^, ^30^, ^43^ which plays a key role in the properties that the hybrid contacts structure exhibits. In more detail, metallization means that the electronic states of graphene under the metal will couple to the metal *d* states^44^ so that metal and graphene will form good ohmic contact with each other. The inset of Figure 1b shows the optical microscope (OM) image of a device based on h‐BN/monolayer MoTe_2_/G as an example. The effect of h‐BN will be discussed in the third section of this work. For MoTe_2_ (Figure 1b), two characteristic Raman‐active modes of A_1g_ and E^1^_2g_ locating at 172 and 235 cm^−1^ were observed. The quenched B^1^_2g_ mode indicates the monolayer thickness of the MoTe_2_ flake.^45^ For G (Figure 1c), the shape and intensity of characteristic 2D (2703 cm^−1^) and G (1580 cm^−1^) peaks indicate the bilayer nature.^46^ In addition, the high quality after transferring and thickness homogeneity of G and MoTe_2_ flakes are further confirmed by the Raman mappings as shown in the inset of Figure 1c.

**Figure 1:**
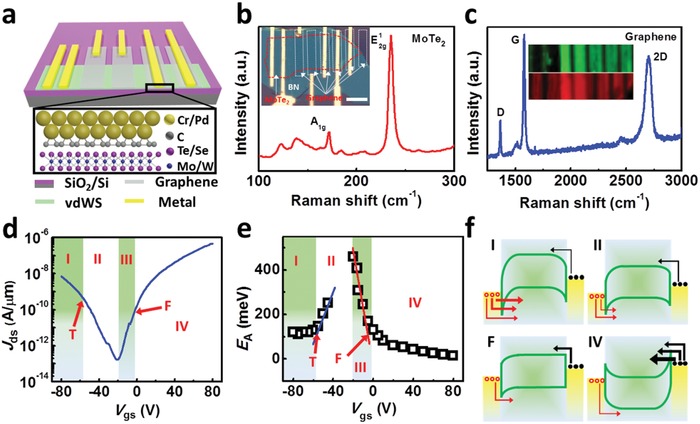
Device structure and the extraction method of charge carrier injection barriers. a) Schematic of the complete device. The inset shows the crystalline structure at the van der Waals semiconductors (vdWS)/graphene/metal region. b) The typical Raman spectra of monolayer MoTe_2_. The inset shows the optical image of the complete device, and the scale bar is 10 µm. c) The typical Raman spectra of bilayer graphene (buffer layer materials or contacts) used in the device. The inset shows the Raman mappings of graphene (upper) and MoTe_2_ (lower) flakes. d) Transfer characteristic of a MoTe_2_ FET with Cr contact at *V*_ds_ = 1.0 V. e) Extracted activation energy for charge carrier injection as a function of *V*_gs_ at *V*_ds_ = 1.0 V. Two transition points where the values stop depending linearly on *V*_gs_ are denoted as T and F, respectively. Four parts of the curves in (d) and (e) are labeled as (I)–(IV), and the corresponding band diagrams are drawn in (f). f) Both *I*_hole_ (red arrows) and *I*_electron_ (black arrows) are made up of either TE current (thermal region) or a combination of tunneling current (TFE or TFE and FE) and TE current (tunneling region). The thickness of lines indicates the strength of the current.

To study the properties of G/M hybrid contacts for ambipolar vdWS, it is necessary to elucidate the mechanism underlying the ambipolar conduction behaviors and re‐establish the corresponding Schottky barrier extraction method. Figure 1d shows the typical transfer characteristic of a MoTe_2_ device with individual Cr contacts, where an obvious ambipolar conduction is observed. Similar conductions can be also found for G and G/M contacts (explained later). It has been demonstrated that this ambipolar conduction behavior can be captured by the Schottky barrier field effect transistor model,^47^ which has been proved to be fully apprehended to the ambipolar behavior observed in CNT,^48^ BP,^47^ MoTe_2_, and WSe_2_ 49 in previous literatures. Actually, the conduction behaviors of SBFET are mainly determined by the voltage tunable Schottky barrier at the contact regions, where both electrons and holes can be injected into/extracted from the channel. And it can be qualitatively understood by the band diagrams depicted in Figure 1f. For a large |*V*_gs_| (regime I/IV), the bands bend strongly down/up at the contact regions allowing electrons (holes) (displayed in black circles and red open circles respectively) to inject into the MoTe_2_ channel only through thermionic emission (TE), while holes (electrons) through field emission (FE), thermionic field emission (TFE), and TE^13^ because of a narrower barrier width. The FE and TFE currents along with the considerable TE currents make the device in apparently on‐state. While decreasing |*V*_gs_|, the bands keep the downward‐bending configuration (the up‐bending configuration may also exist depending on the practical situation), enabling TE and TFE injection modes for holes and sole TE injection mode for electrons. In this situation, both electrons and holes contribute considerably to *J*_ds_ and two specific regimes can be defined based on the relationship between electron (*J*_ds‐e_) and hole (*J*_ds‐h_) currents density: *J*_ds‐e_ < *J*_ds‐h_ for regime II, and *J*_ds‐e_ > *J*_ds‐h_ for regime III. The reduced FE modes and probability for holes and sole TE mode for electrons because of widening barrier width result in relatively small *J*_ds_ making the device in apparently off‐state as shown in Figure 1d. And the minimum point in the drain current density–gate voltage (*J*_ds_–*V*_gs_) curve meets the status where the currents contributed from electrons and holes are the same.

A widely used method to extract the SBH of devices based on vdWS is analyzing the data via the thermionic emission theory and finding the position where flat‐band condition is met. According to the thermionic emission theory, the drain current *I*_ds_ is determined by^47^

(1) $$I_{\mathrm{ds}}\;=\;A^{*}ST^{3/2}\exp\left[-\left(E_{A}-\frac{qV_{\mathrm{ds}}}{n}\right)/\left(k_{B}T\right)\right]$$

where A*, *S*, *k*_B_, *T*, *q*, *V*_ds_ and *n* are the Richardson constant, contact area, Boltzmann constant, temperature, elementary electron charge, voltage bias, and ideality factor coming from image charge, respectively. *E*_A_ is the total activation energy that charge carriers have to overcome to inject into the channel and equals to SBH (*qΦ*_B_) when the energy band of channel material is flat.^13^ Figure 1e shows the extracted *E*_A_ as a function of *V*_gs_ for *V*_ds_ = 1.0 V as an example. The flat‐band condition is usually defined at the point where the values of *E*_A_ stop depending linearly on *V*_gs_ (flat‐band voltage, *V*_F_), which is the transition point of the form of dominant current changing from TE (*I*_TE_) to TFE (*I*_TFE_).^13^ Obviously, two apparent flat‐band points (denoted as F and T) are observed for electrons and holes transport regions, respectively. This phenomenon has been widely observed in devices based on ambipolar vdWS, and the two transition points are usually ascribed to the flat‐band positions of electrons and holes.^18^, ^34^

However, based on the SBFET theory, this band bending scenario is unlikely to happen (Figure 1f). According to the band alignment between MoTe_2_ and Cr (Figure S1, Supporting Information), the band structure of Cr/MoTe_2_/Cr should be consistent with Figure 1f (F) if point F (Figure 1e) corresponds to the flat‐band condition of electrons. At this time, the energy band at holes terminal is bending down. And it will bend down more (Figure 1f (I)–(II)) when gate voltage is further reduced, which implies that there will be no flat‐band condition for holes in the p‐branch. However, flat‐band condition for holes will happen when the gate voltage is increased, which corresponds to the right side of the point F and locates in the electrons branch. Therefore, it is impossible to refer point T (Figure 1e) to the flat‐band condition for holes. And, the flat‐band condition for electrons is going to locate in the holes branch, rather than the electrons branch vice versa. Therefore, one of the two transition points must have another different physical picture. Using the analytical SBFET model we have built on MoTe_2_ and WSe_2_, we conclude that the points F and T represent the normal flat‐band condition of electrons and the transition position at which the dominate injection mechanism of holes changes between TFE and TE, respectively. Obviously, take Figure 1e as an example, the *E*_A_ at points like F refers to the SBH of electrons at flat‐band condition, but the *E*_A_ at points like T does not refer to the SBH of holes. Therefore, we define the *E*_A_ at points like T as effective charge carrier injection barriers (*E*_a_). Note that *E*_a_ does not have a practical meaning like SBH but can also reflect the charge carrier injection ability in ambipolar vdWS due to the crucial role of tunneling in such a device system, i.e., a smaller *E*_a_ represents a better electrical property. In total, for the case shown here, the results extracted via the thermionic emission theory give the SBH of electrons (Region III and IV) and the *E*_a_ of holes (Region I and II), respectively. Note that the analytical process is similar for metal Pd contacts whose schematics including transfer characteristic and band profiles are shown in Figure S3h,i (Supporting Information).

Next we study and compare the properties of the three types of contacts based on the model proposed above. **Figure** **2**a shows the transfer characteristics of a few‐layer MoTe_2_ FET on bare SiO_2_/Si substrate (the OM image of the device is shown in Figure S2a, Supporting Information) with three types of contacts. According to the Schottky–Mott rule and the band alignment between MoTe_2_ and Cr^50^, ^51^ (Figure S1, Supporting Information), MoTe_2_ FET with Cr contacts should display p‐terminal dominated conduction. However, a clear ambipolar with n‐terminal dominated conduction (black line) can be observed because of the FLP effect.

**Figure 2:**
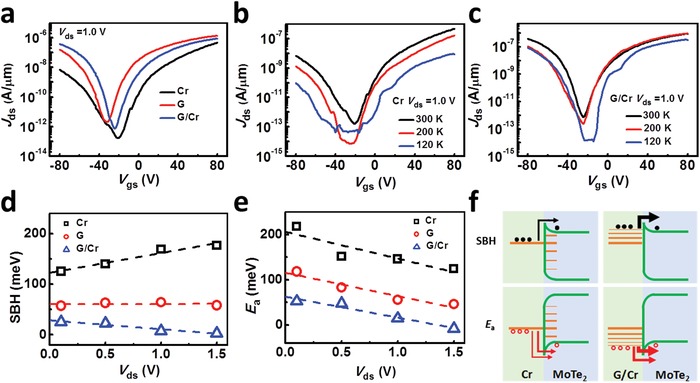
Electrical properties of MoTe_2_ FETs with three types of contacts. a) Transfer characteristics of MoTe_2_ FETs with individual Cr contacts, individual G contacts and G/Cr q‐vdWC at *V*_ds_ = 1.0 V. b,c) Temperature dependence of *J*_ds_–*V*_gs_ curves for MoTe_2_ FETs with individual Cr contacts and G/Cr q‐vdWC at *V*_ds_ = 1.0 V. d,e) Voltage bias dependence of the effective SBH of electrons and *E*_a_ of holes for MoTe_2_ FETs with individual Cr contacts, individual G contacts and G/Cr q‐vdWC. The linear fitting is shown as dashed‐dotted lines. f) Band diagrams of the MoTe_2_ FETs in terms of the experimental results of individual Cr contacts and G/Cr q‐vdWC.

For G contacts (red line), the current density of holes branch at *V*_gs_ = −80 V, increases by more than an order of magnitude compared with Cr contacts (black line). The phenomenon indicates that a more obvious ambipolar behavior is observed because of the strong gate tunable work function of graphene and the van der Waals feature of the MoTe_2_/G interface, which has been found in previous reports.^34^, ^52^ Notably, the ambipolar behavior can be further enhanced by using G/Cr contacts (blue line) which gives rise to additional two times higher than that of G contacts. In addition, if we define *R*_n‐dominated_ = *J*_80V_ */J*_−80 V_ to evaluate the ambipolarity, the values of Cr, G, and G/Cr contacts are 68, 8.5, and 2.3, respectively. Apparently, G/Cr electrode shows a most obvious ambipolar behavior. A number of ab initio calculation works have predicted that the inserting of van der Waals 2D material layers, like h‐BN and graphene, will break the hybridization between metal and vdWS channels (i.e., reduce the metal induced gap states, MIGS) and enable the van der Waals features.^29^, ^30^ Especially for vdWS/G/M system, it has shown that the G interlayer will form a hybrid system with the metal. The introduction of metal would induce a small amount of metal induced gap states within the bandgap of vdWS channels at the G/M hybrid contacts region.^29^ However, it has been demonstrated that the work function of graphene hybridized to a metal do can be tuned by a remote gate.^29^, ^53^ Although the gate controllability of G/M hybrid contacts would be weakened because of the introduction of metal, the tunable intensity should be better than the individual metal contacts. This can be demonstrated by comparing the gate‐dependent SBH extracted from transistors with G/Cr hybrid contacts and Cr contacts (Figures S2f and S1e): it changes ≈85.3 meV in the case of G/Cr hybrid contacts (Figure S2f) against the ≈51.4 meV in the individual Cr contacts (Figure 1e) under Δ*V*_gs_ = −4 V from *V*_gs_ = −20 to −16 V. In total, G/M hybrid contacts enhance the carrier injection from metal into channel, which we believe is actually the reason of the enhanced ambipolar properties observed here. To differentiate from the absence of MIGS at the vdWS/G interface, we name G/M hybrid contacts of quasi‐van der Waals contacts (q‐vdWC).

The features of q‐vdWC can also be seen by comparing the temperature‐dependent transfer curves of individual metal contacts, individual G contacts, and G/Cr q‐vdWC (Figure 2b,c, Figure S2c, Supporting Information). Compared with individual Cr contacts, the G/Cr q‐vdWC shows comparable gate tunability of Fermi level with G contacts; it has been proven that the G/Cr q‐vdWC and individual G contacts both have a stronger tunneling capacity featured with the almost independency of *J*_ds_ on *T* at high *V*_gs_. To get a more convincing conclusion, we also analyze the charge carrier injection abilities of the three types of contacts via the quantitative model mentioned above. Figure 2d and e show the SBH of electrons (*Φ*_e_) and *E*_a_ of hole (*E*_a‐hole_), respectively (Figure S2e–g and Table S1, Supporting Information). The smallest *Φ*_e_ and *E*_a‐hole_ of ≈28.3 and 62.6 meV are found in G/Cr q‐vdWC, which are about 4.3 and 3.3 times smaller than that of Cr contacts. Although the tunneling ability seems comparable between G/Cr with individual G contacts if only according to the dependency of *J*_ds_–T relationship (Figure S2d, Supporting Information), the quantitative data show that the smallest *Φ*_e_ and *E*_a‐hole_ of the former (≈28.3 and 62.6 meV) about 2.1 and 1.8 times smaller than that of the latter (≈60.2 and 115.4 meV) (Table S1, Supporting Information). Compared with individual G contacts, the existence of metal (which has much higher density of states) will induce orbital perturbation at the interface between graphene and underneath channel. Furthermore, there will be charge accumulation and depletion regions at the interface, which will lead to charge distribution and further dipole formation resulting in the decrease of SBH.^30^, ^31^ Therefore, the device with G/Cr q‐vdWC shows better electrical characteristics than that with G contacts.

The advantages of G/M q‐vdWC can be qualitatively understood by the band diagrams depicted in Figure 2f. Due to the large density of MIGS at vdWS/M interface, the Fermi level is strongly pinned (left column). Only TE and/or TFE can happen for large gate voltages. On the other hand, due to the quasi‐van der Waals feature of the interface between G under the metal and vdWS, the Fermi level of G/M can be tuned strongly approaching to the conduction band minimum (CBM) and the valence band maximum (VBM) at positive (right up panel) and negative (right bottom panel) gate voltages, respectively. As a consequence, direct tunneling can happen for both electrons and holes. Furthermore, graphene under the metal will be doped by charge transfer from metal with high density of states.^54^, ^55^, ^56^ Thus, there will be a smaller energy difference between the work function of graphene and the conduction band minimum or the valence band maximum of channel material before the two materials come into contact under the same electrical conditions,^29^ which is the reason that G/M contacts is superior to the individual graphene contacts (Figure S2i, Supporting Information). As a result, smaller SBH and *E*_a_ will be induced compared with individual metal and individual graphene contacts.

The above phenomena can also be generalized to other metals and ambipolar vdWS. The results of MoTe_2_ FETs with Pd as metal electrodes and WSe_2_ FETs with Cr as metal electrodes are shown in Figures S3 and S4 (Supporting Information), respectively. For MoTe_2_ FET with individual Pd contacts, the device shows p‐terminal dominated ambipolar conduction that can be seen in Figure S3c,d (Supporting Information). Further, if we define *R*_p‐dominated_ = *J*_−80 V_/*J*_80V_ to evaluate its ambipolarity in a quantitative way, the values for Pd contacts at 300, 220, and 120 K are calculated to be 6.3, 3, and 10.5, respectively, more obviously indicating the p‐dominated feature. While using G/Pd q‐vdWC, the current density of electron terminal is improved making the device act as n‐terminal–dominated ambipolar conduction (Figures S3e and S6g, Supporting Information). Furthermore, smaller SBH (130.5 meV) for holes and *E*_a_ (54.3 meV) for electrons are extracted comparing to the values of individual Pd contacted MoTe_2_ FET (SBH 166.4 meV and *E*_a_ 111.1 meV). Similarly, by using G/Cr q‐vdWC, the injection of holes to WSe_2_ channel is remarkably enhanced (Figure S4c, Supporting Information) and the extracted SBH for electrons is reduced to 58 meV (Figure S4g, Supporting Information). In total, we conclude that G/M q‐vdWC combine the advantages of individual metal contacts (high density of states) and individual G contacts (large Fermi level tunability), making it a promising strategy to improve the contact quality for vdWS FETs.

It has been shown that the dielectric environment, like the substrate used, plays a crucial role in the Schottky barrier of vdWS FETs due to the atomically thin thickness.^57^ Hence, we then investigate the influence of substrate on the contact performance of G/M q‐vdWC. Here, we primarily focus on the effect of h‐BN substrate on MoTe_2_ FET with individual Pd contacts and G/Pd q‐vdWC. The Raman spectra of the two devices are shown in Figure 1 and Figure S3 (Supporting Information), respectively, indicating that the two devices are comparable. **Figure** **3**a,b shows the differences in *J*_ds_–*V*_gs_ curves between SiO_2_/Si and h‐BN substrate of devices with individual Pd contacts and G/Pd q‐vdWC, respectively. For the former, it reveals that the current density (*J*_ds_) of hole‐terminal decreases while the electron‐terminal increases when putting MoTe_2_ channel on h‐BN substrate. This is because the decreased effective work function of MoTe_2_ arisen from h‐BN inducing n‐doping results in the reduced *E*_a_ of electrons and increased SBH of holes, which agrees well with previous report.^57^

**Figure 3:**
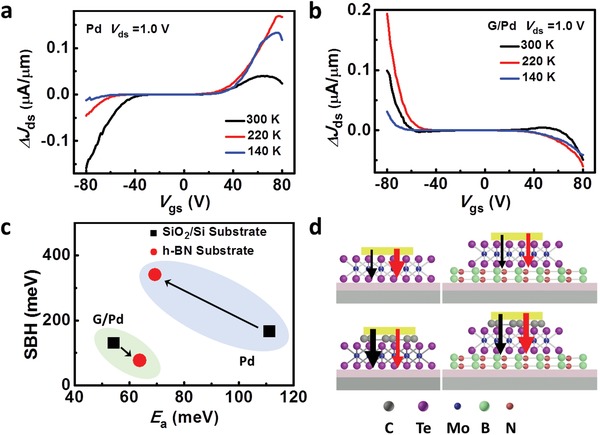
Influence of substrate on G/M q‐vdWC. Current density difference‐gate voltage curves of MoTe_2_ devices with a) individual Pd contacts and b) G/Pd q‐vdWC between h‐BN substrate and SiO_2_/Si substrate. c) Summarization of *Φ*_h_ and *E*_a‐electron_ values of MoTe_2_ FETs with individual Pd contacts on SiO_2_/Si and h‐BN substrates, and G/Pd q‐vdWC on SiO_2_/Si and h‐BN substrates. d) Charge carrier injection diagrams for MoTe_2_ FETs with four kinds of structures. The black and red arrows represent the conductions of electrons and holes, respectively, with the thickness indicating the current strength.

However, an opposite change was observed for G/Pd q‐vdWC as shown in Figure 3b. The SBH and *E*_a_ were extracted and drawn in Figure S6a–d and the exact values at zero bias are summarized in Table S1 in the Supporting Information. Here the SBH and *E*_a_ refer to the SBH for holes (*Φ*_h_) and thermal‐assisted activation energy for electrons (*E*_a‐electron_). The influence of h‐BN on the charge carrier injection barriers are summarized in Figure 3c. For *Φ*_h_, the value of individual Pd contacts increases more than twice (from 166.4 to 340.5 meV), while the value of G/Pd q‐vdWC decreases ≈58% (from 130.5 to 76.9 meV) when devices are fabricated on h‐BN substrate. On the other hand, for *E*_a‐electron_, the value of individual Pd contacts decreases ≈62% (from 111.1 to 69.3 meV), while the value of G/Pd q‐vdWC increases about 10 meV (from 54.3 to 63.8 meV). This means that the phenomenal electron doping effect induced by h‐BN is invalid for G/M q‐vdWC‐based MoTe_2_ FET, which is attributed to the screening effect of graphene. It has been demonstrated that there will be orbitals coupling between metal contact, channel, and substrate, not just between metal contact and channel.^58^ In addition, the electrical properties of ambipolar devices with metal contacts are seriously affected by the substrate due to the change of orbital coupling while changing the substrates.^48^ However, inserting a layer of graphene between metal and channel can break the coupling and eliminate the influence from the substrate. Consequently, the electrical properties of devices with G/M q‐vdWC are independent of substrate effect.

The schematic diagrams of influences of h‐BN substrate on the transport properties for the two contacts are depicted in Figure 3d. Here black and red arrows represent the injections of electrons and holes, respectively. Note that we do not distinguish the vertical and lateral transports of charge carriers for simplicity.^28^, ^29^ The opposite change tendency and the smaller changes that G/Pd q‐vdWC experiences indicate that the inserted graphene layer not only brings in a quasi‐van der Waals interface, but also enables immunity to the influence from the substrate. This synergistic effect may make G/M q‐vdWC a promising choice to achieve the Schottky–Mott limit.^24^

To elucidate the Fermi level unpinning effect of G/M q‐vdWC, the location diagram of various contact types relative to the *E*_c_ and *E*_v_ of MoTe_2_ by their extracted SBH and *E*_a_ (Table S1, Supporting Information) are displayed in **Figure** **4**a and b, respectively. For Cr and G/Cr contacts, the distance between data points and *E*_c_ (*E*_v_) is proportional with extracted SBH in Figure 4a (*E*_a_ in Figure 4b). However, for Pd and G/Pd contacts, the distance between data points and *E*_c_ (*E*_v_) is proportional with extracted *E*_a_ in Figure 4b (SBH in Figure 4a). Take the red open circle of Figure 4a as an example, the distance between its location and *E*_c_ is proportional to the extracted SBH (28.3 meV in Table S1, Supporting Information) of the MoTe_2_ FET with G/Cr contacts. For individual Cr and G/Cr contacts, the devices show n‐terminal dominated conduction. On the contrary, for individual Pd and G/Pd contacts, the devices show p‐terminal dominated conduction. While using G/M q‐vdWC, the Fermi level of the hybrid system approaches closer to the band edges, which is a sign of Schottky–Mott rule. In addition, due to the strong Fermi level tunability, smaller *E*_a_ can be achieved at the same time as shown in Figure 4b.

**Figure 4:**
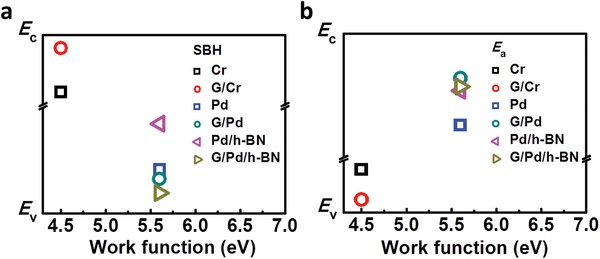
The relative locations of various electrode types to the *E*_c_ and *E*_v_ of MoTe_2_ using the extracted SBH and *E*_a_ to elucidate the Fermi level unpinning effect of G/M q‐vdWC. Dependence of extracted a) SBH and b) *E*_a_ from Table S1 (Supporting Information) on the work function of metals used. *E*_c_ and *E*_v_ refer to the conduction band minimum and the valence band maximum of MoTe_2_, respectively. The work functions of Cr and Pd are taken to be 4.5 and 5.6 eV, respectively. For Cr and G/Cr contacts, the distance between data point and *E*_c_ (*E*_v_) of MoTe_2_ is proportional with SBH in Figure 4a (*E*_a_ in Figure 4b). However, for Pd and G/Pd contacts, the distance between data points and *E*_c_ (*E*_v_) is proportional with extracted *E*_a_ in Figure 4b (SBH in Figure 4a).

Finally, we investigate the photoresponse properties of devices using G/M q‐vdWC. Here, a 473 nm laser with variable power densities of 2.55–248.3 mW cm^−2^ was used as the light source. **Figure** **5**a,c shows the *I*_ds_–*V*_gs_ curves under dark and illuminated states of G/Pd q‐vdWC MoTe_2_ and G/Cr q‐vdWC WSe_2_ phototransistors. For the former, the transfer curves shift toward negative *V*_gs_ while increasing the laser power, which is attributed to the photogating effect.^4^, ^59^ The photogating effect is relative stronger for small *V*_gs_ evidenced by the steeper dependence of photocurrent (*I*_ph_, defined as *I*_illumanted_ − *I*_dark_) on laser power (Figure S7a, Supporting Information) and the positive slopes of Responsivity (*R*)‐laser power density relations (Figure 5b). Here, *R* = *I*_ph_/*PS*, where *P* and *S* are the laser power densities and the effective illumination area, respectively. On the other hand, obvious photoresponse capability along with photogating effect can be found for WSe_2_ phototransistor (Figure 5c,d). The devices with G/M q‐vdWC show nonmonotonic dependence of R on laser power, which we attribute to the reduced barriers for both holes and electrons (see Note S1 in the Supporting Informationfor more details).

**Figure 5:**
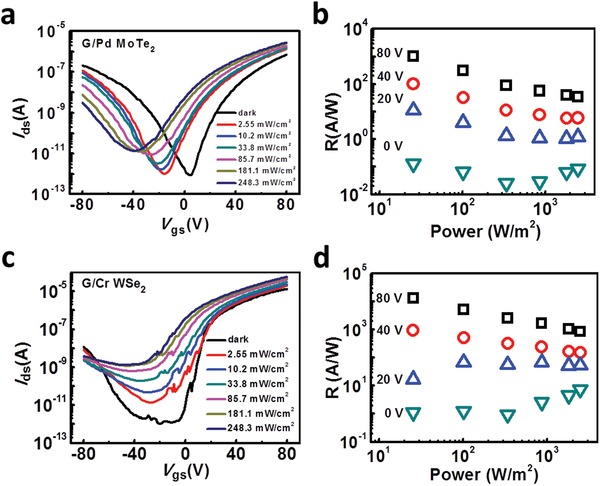
Photoresponse properties of phototransistors with G/M q‐vdWC. Transfer curves under dark and illuminated states of a) MoTe_2_ phototransistor with G/Pd q‐vdWC and c) WSe_2_ phototransistor with G/Cr q‐vdWC. The laser power‐dependent responsivity of MoTe_2_ and WSe_2_ phototransistors at various gate voltages are shown in (b) and (d), respectively.

Similar phenomena can be observed in other devices (Figures S8 and S9, Supporting Information). To quantitatively evaluate the performance of the devices, we calculate the *R* and Detectivity (D*, defined as *I*_ph_/*P*(2*qSI*_dark_)^1/2^ by assuming that the dark current dominates the shot noise). Figure 5b,d and Figure S7c,f (Supporting Information) show the results, respectively. For MoTe_2_ phototransistor, the highest *R* and D* are 1012 A W^−1^ and 1.02 × 10^10^ jones, respectively, achieved at *V*_gs_ = 80 V and *P* = 2.55 mW cm^−2^. As for WSe_2_ phototransistor, the highest *R* of 1.33 × 10^4^ A W^−1^ and D* of 2.98 × 10^10^ jones can be obtained at *V*_gs_ = 80 V and *P* = 2.55 mW cm^−2^. These data are among the highest performances of photo‐detectors based on pure MoTe_2_ and WSe_2_.^34^, ^35^, ^36^, ^37^, ^38^, ^39^, ^40^

In summary, we systematically study the properties of graphene/metal quasi‐van der Waals contacts. First, a re‐established model to extract the barriers of charge carrier injection in ambipolar van der Waals semiconductors is proposed. The widely observed two transition points phenomenon is attributed to the existence of normal flat‐band condition and the transition position of dominate injection mechanism between TFE and TE, respectively. Based on this model, we find graphene/metal quasi‐van der Waals contacts can combine the advantages of individual metal and graphene van der Waals contacts. With high density of states and quasi‐van der Waals interfaces, these contacts show significantly lower Schottky barrier and TFE activation energy regardless of the metal and substrate types. Besides, we find the graphene/metal quasi‐van der Waals contacts show immunity to the influence of substrate and Fermi level pinning effect. All these features make this contacts structure a promising strategy to enhance the advantages of ambipolar van der Waals semiconductors. In addition, MoTe_2_ and WSe_2_ phototransistors with this special contacts exhibit very high photoresponse performances with *R* as high as 1012 and 1.33 × 10^4^ A W^−1^, respectively. The contact strategy we propose here may be an alternative choice that can be generalized to other van der Waals semiconductors to approach the Schottky–Mott limit.

## Experimental Section

*Device Fabrication*: The graphene (as electrodes or buffer layer materials) and vdWS flakes (MoTe_2_ and WSe_2_) were first exfoliated from the bulk crystal (99.995%, HQ Graphene) onto the clean 300 nm SiO_2_/Si substrates using the standard scotch method. For the devices with h‐BN substrates, exfoliated vdWS flakes were first transferred on h‐BN layer by dry‐transfer technic. Standard electron‐beam lithography (EBL) followed with reactive ion etching (RIE) process was used to get graphene stripe arrays with about 2 µm spacing. After that, the graphene stripe arrays were further transferred onto the pre‐exfoliated vdWS flakes. Finally, a thin layer of Cr/Au (10/50 nm) or Pd/Au (10/50 nm) was defined and deposited at the specified position to form devices with three types of contacts on the same vdWS flakes.

*Materials Characterization and Device Measurement*: The morphology, material quality, and thickness characterizations were performed by OM (Olympus BX51 M), Raman spectroscopy (Renishaw InVia, 532 nm excitation laser), and AFM (Veeco Multimode), respectively. The electrical transport measurements were carried out on a probe station (Lakeshore, TTP4) equipped with a vacuum pump, a flow cryostat, and a semiconductor characterization system (Keithley 4200). The optoelectronic properties were measured using a 473 nm laser with a diameter of 3 mm (RGBLase). The light intensity was varied using a continuous attenuator.

## Conflict of Interest

The authors declare no conflict of interest.

## Supporting information

Supplementary
