# Supplementary material for: Controlling Injection Barriers for Ambipolar 2D Semiconductors via Quasi‐van der Waals Contacts
Source: Adv Sci (Weinh). 2019 Apr 19;6(11):1801841. doi: 10.1002/advs.201801841 (PMC6548948; doi:10.1002/advs.201801841)
Supplement: Supplementary file 1 — Supplementary [file ADVS-6-1801841-s001.pdf]

Copyright WILEY-VCH Verlag GmbH & Co. KGaA, 69469 Weinheim, Germany, 2019.

ADVANCED SCIENCE
Open Access

# Supporting Information

for *Adv. Sci.,* DOI: 10.1002/advs.201801841

## Controlling Injection Barriers for Ambipolar 2D Semiconductors via Quasi-van der Waals Contacts

*Junjun Wang, Feng Wang, Zhenxing Wang, Ruiqing Cheng, Lei Yin, Yao Wen, Yu Zhang, Ningning Li, Xueying Zhan, Xiangheng Xiao,* Liping Feng,* and Jun He**

# Supporting Information

# Controlling Injection Barriers for Ambipolar Two-Dimensional Semiconductors via Quasi-van der Waals Contacts

Junjun Wang, Feng Wang, Zhenxing Wang, Ruiqing Cheng, Lei Yin, Yao Wen, Yu Zhang, Ningning Li, Xueying Zhan, Xiangheng Xiao,* Liping Feng,* and Jun He*

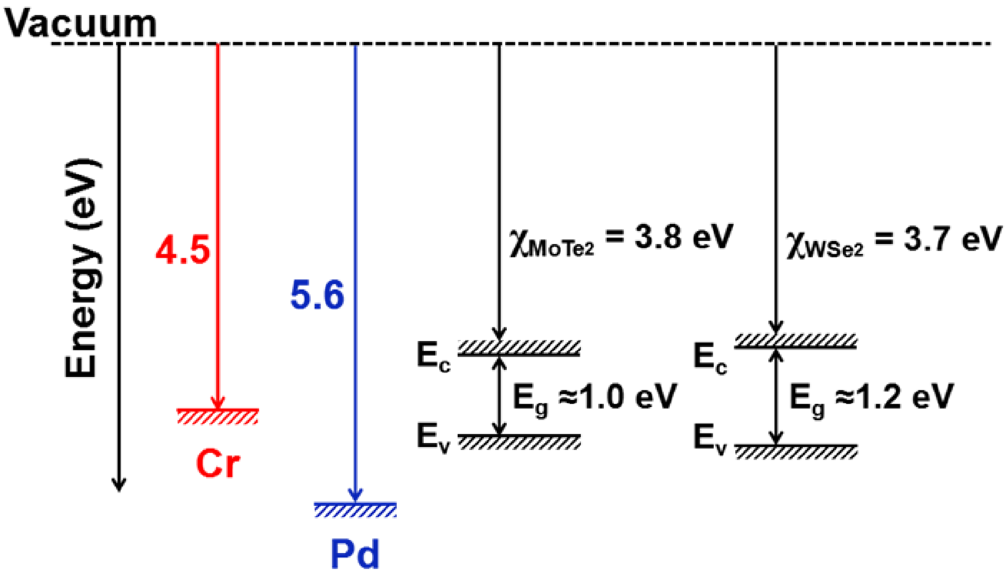


**Figure S1.** Band alignments of Cr, Pd, $MoTe_2$ and $WSe_2$.

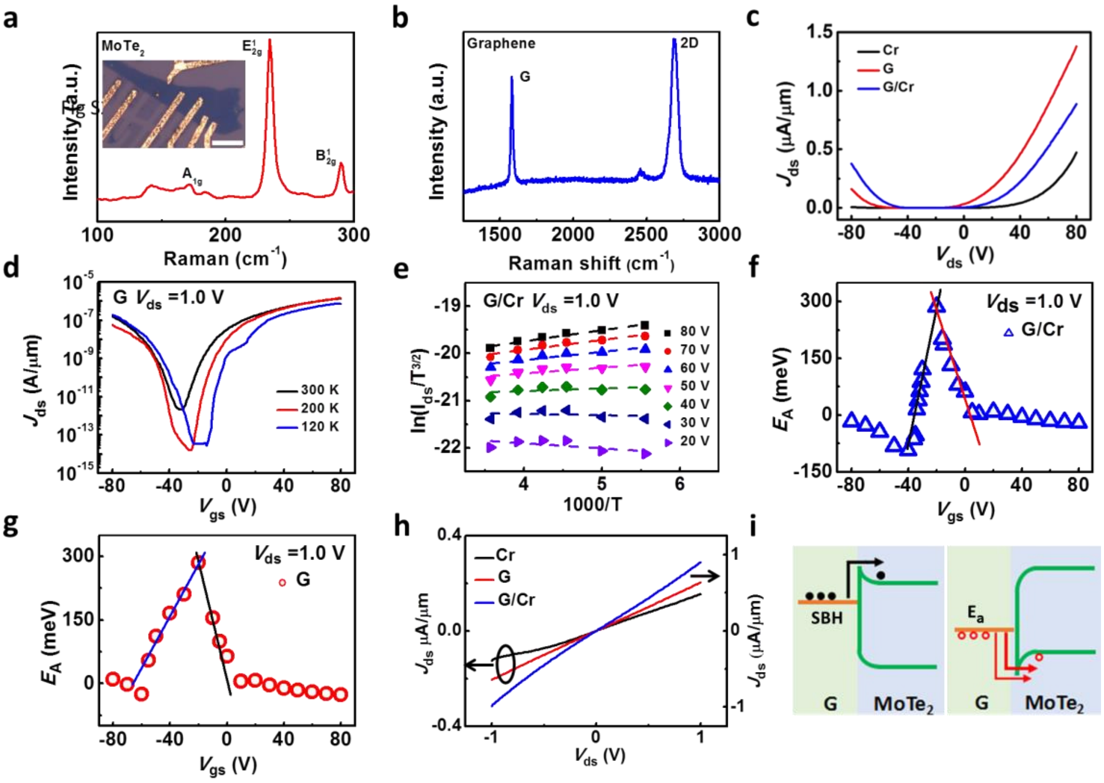


**Figure S2.** Electrical properties of few-layer $MoTe_2$ FETs. Raman spectra of few-layer $MoTe_2$ a) and graphene b). The inset in a) shows the optical image of the $MoTe_2$ FET with pure Cr contacts, pure G contacts, and G/Cr q-vdWC. The scale bar is 10 μm. c) Transfer characteristics in linear coordinates of $MoTe_2$ FETs with individual Cr contacts, individual G contacts and G/Cr q-vdWC at $V_{ds}$ = 1.0 V. d) Temperature dependence of $J_{ds}$-$V_{gs}$ curves for $MoTe_2$ FETs with pure G contacts at $V_{ds}$ = 1.0 V. e) The Arrhenius plot of $\ln(I_{ds}/T^{3/2})$ vs. 1000/T for G/Cr q-vdWC at $V_{ds}$ = 1.0 V. f-g) The extracted $E_A$ as a function of $V_{gs}$ for $MoTe_2$ FETs with G/Cr q-vdWC and pure G contacts, respectively. h) Out-put characteristics at $V_{gs}$ = 80 V for three types of contacts. i) Band diagrams of $MoTe_2$ FETs with pure G contacts at a certain $V_{gs}$. Note that the two band structures (left and right) is in different $V_{gs}$ conditions.

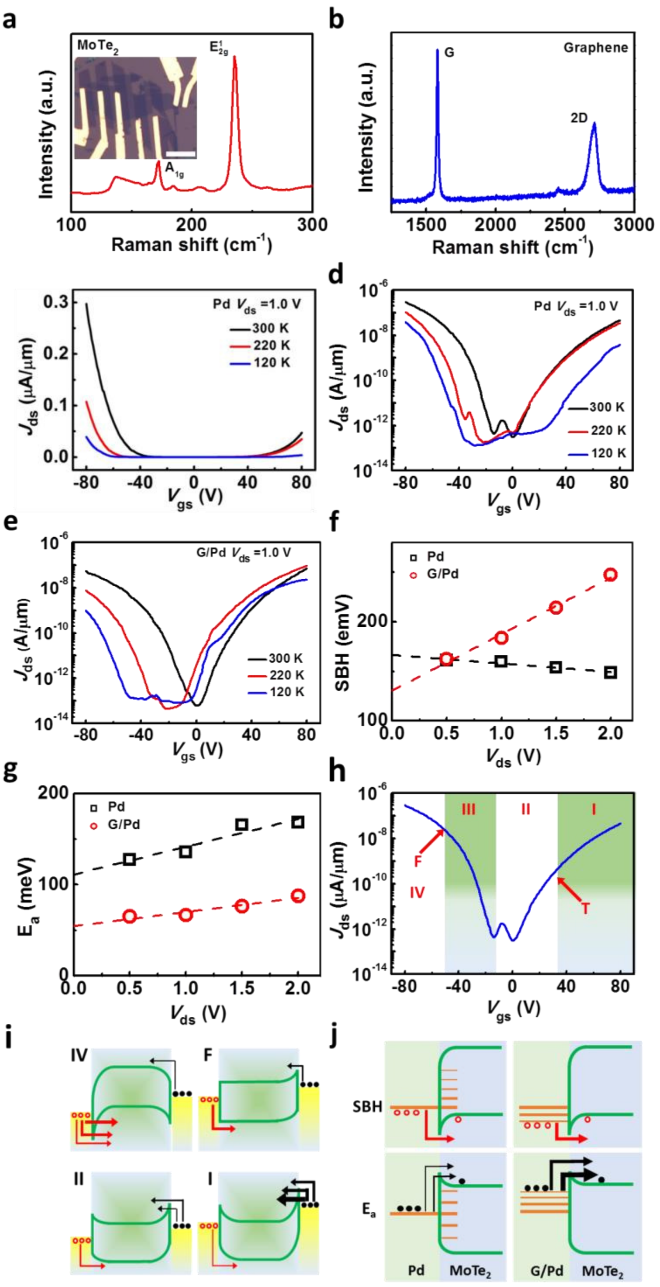


**Figure S3.** Electrical properties of monolayer $MoTe_2$ FETs. Raman spectra of monolayer $MoTe_2$ a) and few-layer G b). The inset in a) shows the optical image of the $MoTe_2$ FET with pure Pd contacts, pure G contacts, and G/Pd q-vdWC. The scale bar is 10 μm. Temperature dependence of $J_{ds}$-$V_{gs}$ curves for $MoTe_2$ FETs with pure Pd contacts in linear c) and logarithmic d) coordinates and e) G/Pd q-vdWC at $V_{ds}$ = 1.0 V. The extracted f) SBH of holes and g) $E_a$ of electrons for the two contacts. h) Transfer characteristic of a $MoTe_2$ FET with Pd contacts at $V_{ds}$ = 1.0 V. i) The band alignments of Pd contacts. j) Band diagrams of $MoTe_2$ FETs with the two contacts, respectively.

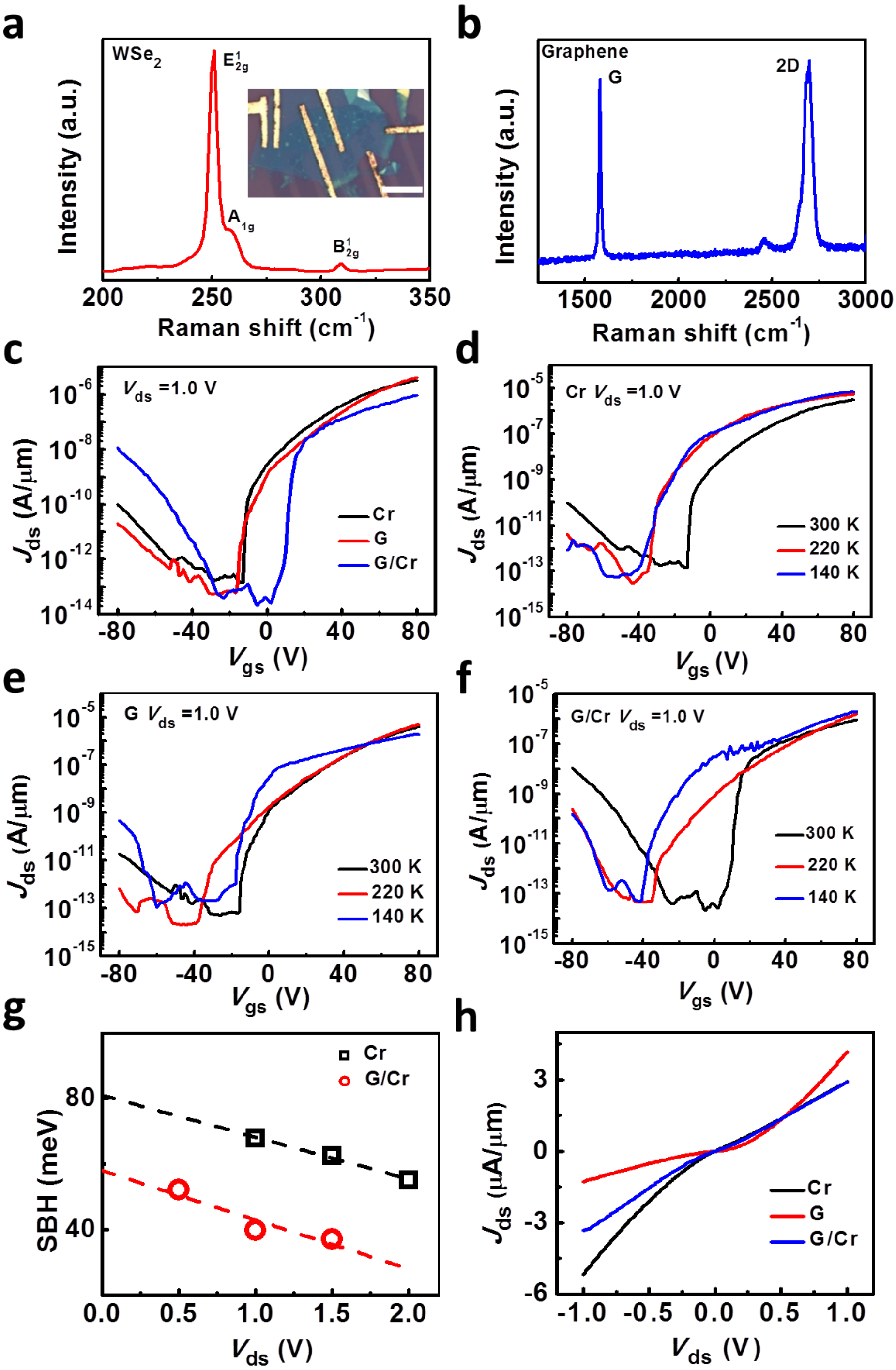


**Figure S4.** Electrical properties of few-layer $WSe_2$ FETs. Raman spectra of a) few-layer $WSe_2$ and b) graphene. The inset in a) shows optical image of the $WSe_2$ FETs with pure Cr contacts, pure G contacts and G/Cr q-vdWC. The scale bar is 10 µm. c) Transfer characteristics of the few-layer $WSe_2$ FETs with three types of contacts at $V_{ds}$ = 1.0 V. d-f) Temperature dependent transfer characteristics for devices with three types of contacts at $V_{ds}$ = 1.0 V. g) The extracted SBH of electrons for pure Cr contacts and G/Cr q-vdWC. The linear fitting are shown as dashed-dotted lines. h) Out-put characteristics under a certain gate voltage for the three types of contacts at $V_{gs}$ = 80 V.

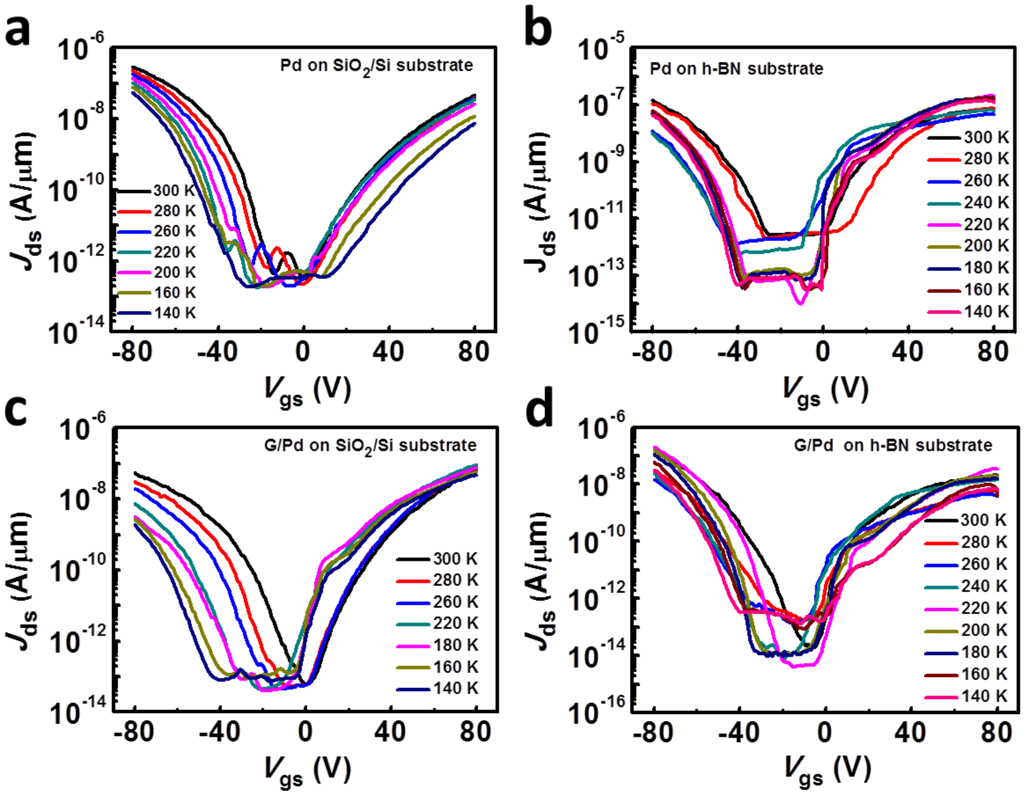


**Figure S5.** Electrical properties of monolayer $MoTe_2$ FETs with different types of contacts fabricated on $SiO_2$/Si and h-BN substrates. The transfer curves at various temperatures for monolayer $MoTe_2$ FET with a-b) pure Pd contacts and c-d) G/Pd q-vdWC.

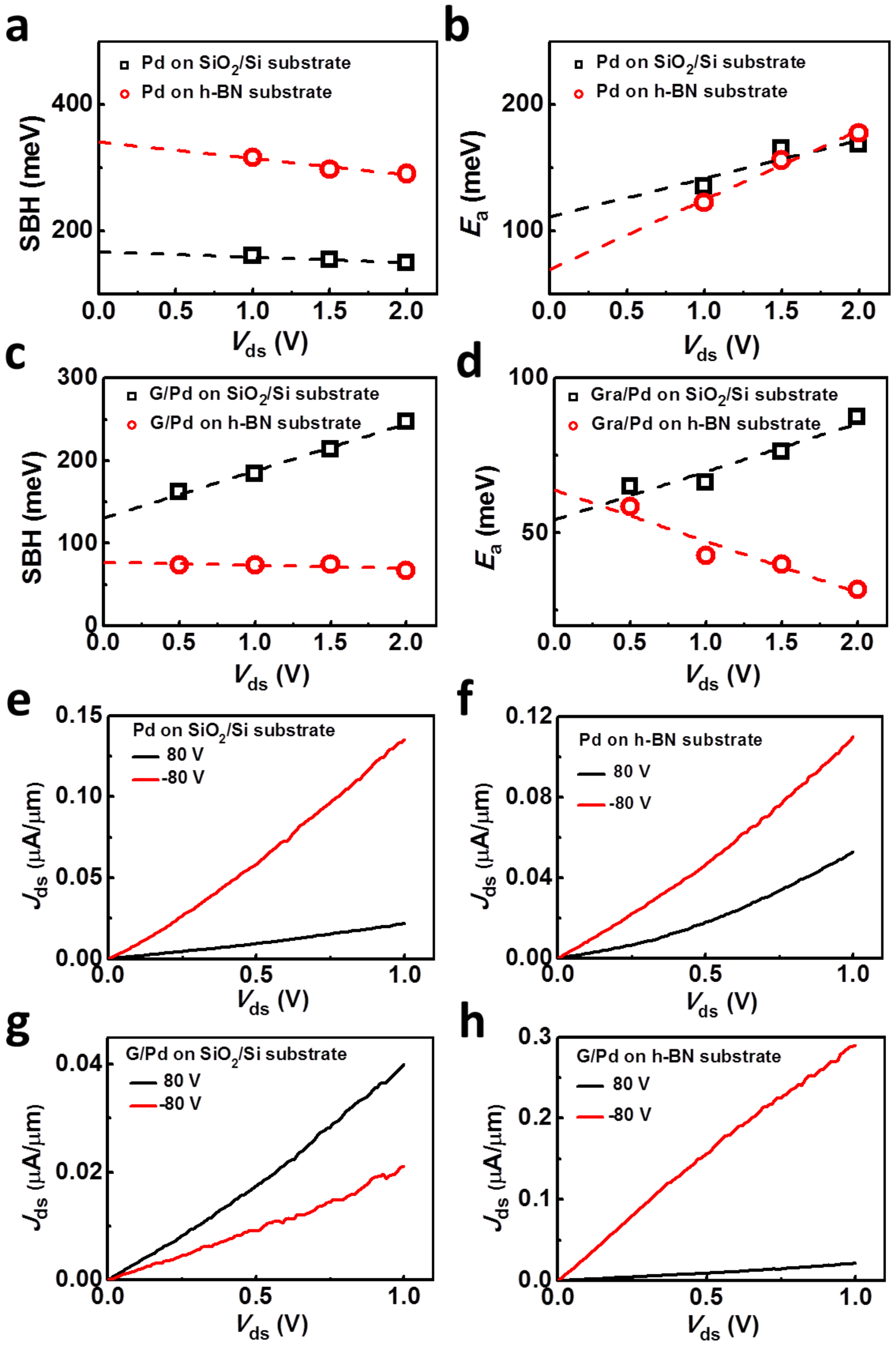


**Figure S6.** Electrical properties of monolayer $MoTe_2$ FETs. Extracted a) SBH and b) $E_a$ for pure Pd contacts fabricated on $SiO_2$/Si and h-BN substrates, respectively. Extracted c) SBH and d) $E_a$ for G/Pd q-vdWC fabricated on $SiO_2$/Si and h-BN substrates, respectively. The corresponding out-put characteristics of the two contact structures at certain gate voltages are shown in e-h).

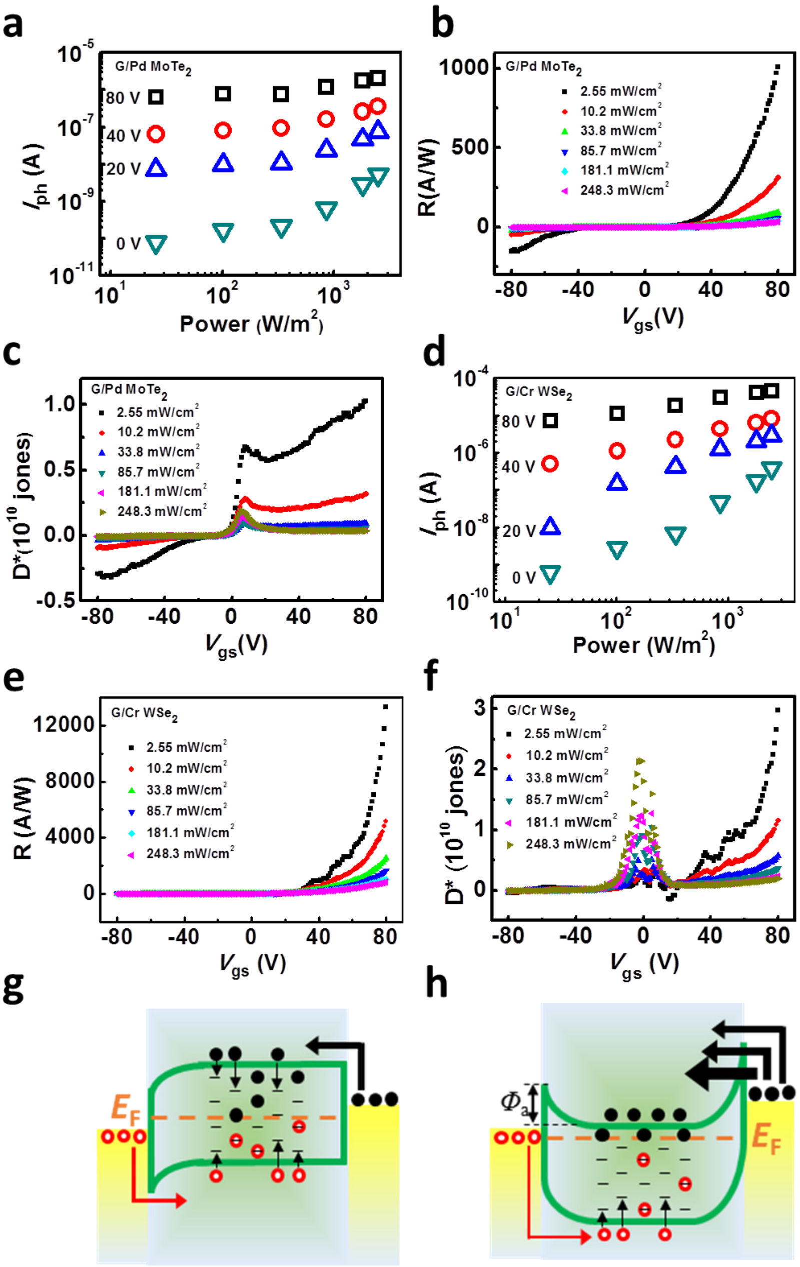


**Figure S7.** Photoresponse properties of ambipolar vdWS phototransistors with G/M q-vdWC. The a) photocurrent b) responsivity and c) detectivity as a function of $V_{gs}$ at various illuminations for $MoTe_2$ phototransistor with G/Pd q-vdWC. The corresponding values for $WSe_2$ phototransistor with G/Cr q-vdWC are shown in d), e) and f). Photocurrent generation mechanism schematics of devices band profiles for g) $V_{gs} = 0$ V and h) $V_{gs} > 0$ V. Up and down black small arrows denote the carrier trapping process. Black dots and red open circles represent electrons and holes, respectively. Black short lines denote electron or hole trap states. The thickness of lines indicates the strength of the current.

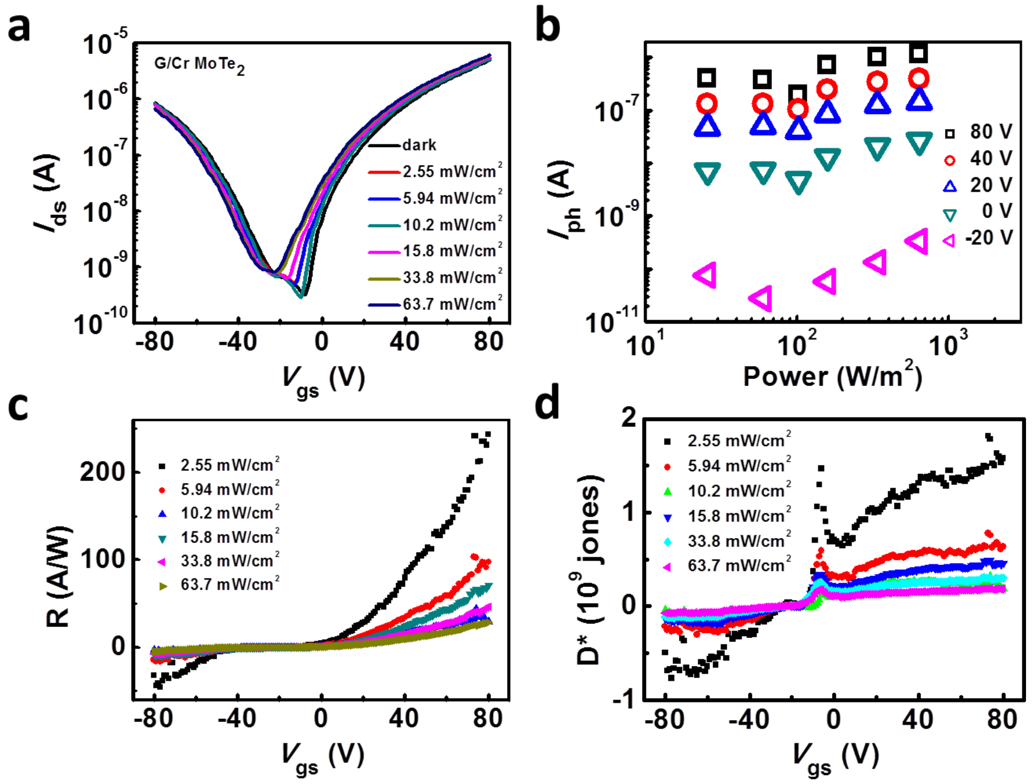


**Figure S8.** Photoresponse properties of few-layer $MoTe_2$ phototransistor with G/Cr q-vdWC. a) Transfer curves of the phototransistor under dark and different illuminated states. b) The photocurrent-laser power curves at different $V_{gs}$. The c) responsivity and d) detectivity as a function of $V_{gs}$ at various illumination states.

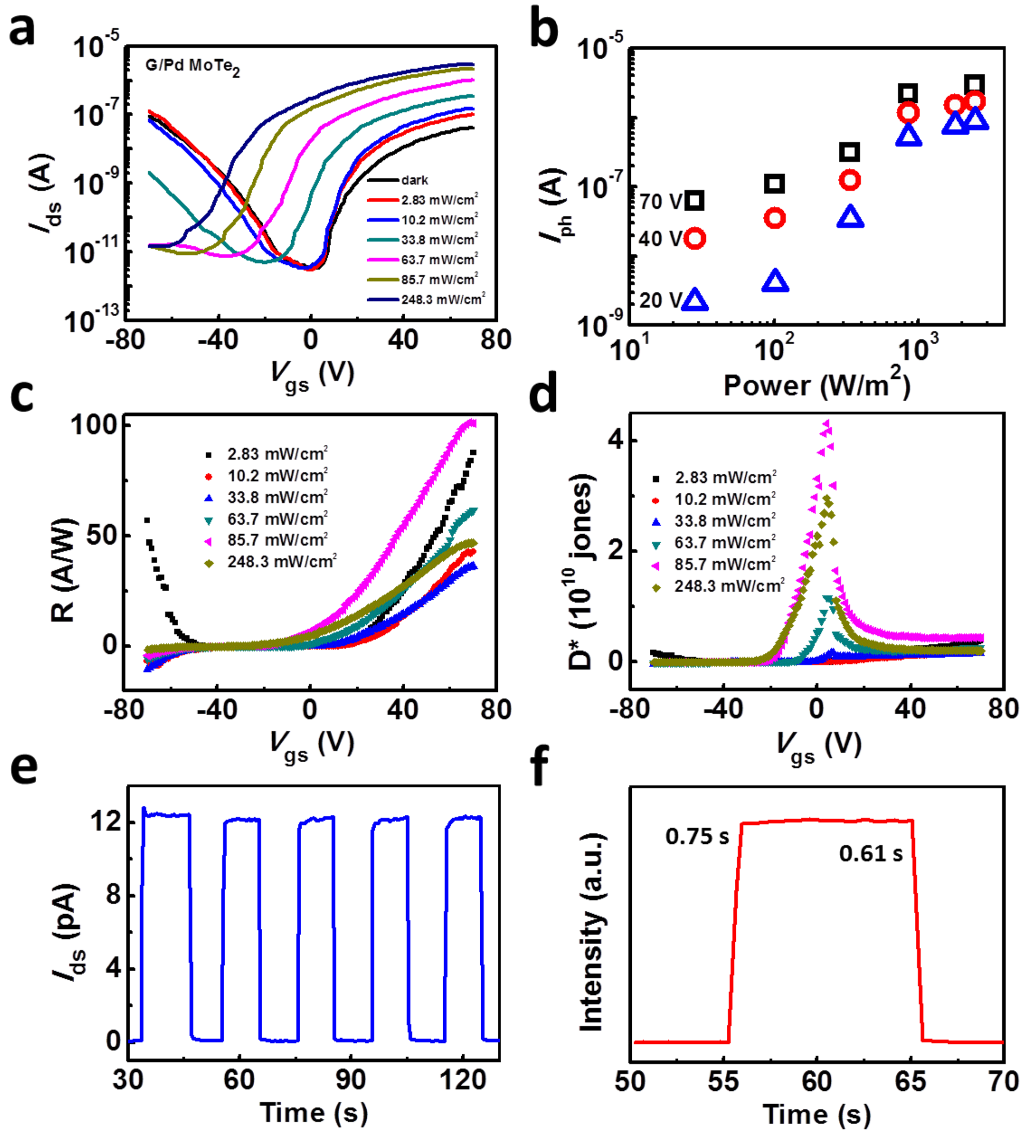


**Figure S9.** Photoresponse properties monolayer $MoTe_2$ phototransistor with G/Pd q-vdWC fabricated on h-BN substrate. a) Transfer curves of the phototransistor under dark and different illuminated states. b) The relationships between photocurrent and laser power at various $V_{gs}$. The c) responsivity and d) detectivity as a function of $V_{gs}$ at various illuminated states. e) The long-term stability of the phototransistor. f) The time-resolved photoresponse showing the rise and decay time about 0.75 s and 0.61s, respectively.

**Table S1.** Summary of the substrate, electrode, channel materials of ambipolar vdWS FETs, and extracted Schottky barrier heights and thermal-assisted activation energies. The null values come from the absence of the transition conditions in the measurement range.

| Device | Substrate | Contacts | SBH (meV) | $E_a$ (meV) |
|---|---|---|---|---|
| #1 $MoTe_2$ FET | $SiO_2$/Si | Cr | 122.5 (electron) | 205.9 (hole) |
| | | G | 60.2 (electron) | 115.4 (hole) |
| | | G/Cr | 28.3 (electron) | 62.6 (hole) |
| #2 $MoTe_2$ FET | $SiO_2$/Si | Pd | 166.4 (hole) | 111.1 (electron) |
| | | G/Pd | 130.5 (hole) | 54.3 (electron) |
| #3 $MoTe_2$ FET | h-BN | Pd | 340.5 (hole) | 69.3 (electron) |
| | | G/Pd | 76.9 (hole) | 63.8 (electron) |
| #4 $WSe_2$ FET | h-BN | Cr | 81 (electron) | |
| | | G/Cr | 58 (electron) | |

**Supplementary Note 1**

**Photocurrent generation mechanism in $MoTe_2$ ($WSe_2$ FET):**

Photoresponse is an important parameter to characterize the optoelectronic performance of devices. For 2D materials, it has been found that the contact properties play a vital role on the photoresponse performances. Generally, photoresponse is closely relevant to trap states and lifetime of photogenerated carriers. [1,2]Trap states with higher energies than Fermi level ($E_F$) are able to capture electrons (electron traps), and those with lower energies than $E_F$ are able to capture holes (hole traps).

For our devices, at $V_{gs} > 0$ V (**Figure S7**h), $E_F$ is located close to the conduction band ($E_c$), and the concentration of free electrons in $E_c$ is high whereas the concentration of free holes in valence band ($E_v$) is low. The devise is in on-state. Under dark condition, electron traps nearby $E_F$ are mostly occupied whereas holes traps are nearly left empty. Under illumination, electron-hole pairs are generated and holes are subsequently trapped which prolongs the electron lifetime. However, at this condition, a barrier ($\Phi_a$) for electrons exists at the drain metal contacts, which

increases the probability of electron-hole recombination[3]. At higher laser power, a large number of carriers in the channel are recombined resulting in a negative dependence of responsivity on laser power, similar with a traditional photoconductor.

At $V_{gs}$ = 0 V (Figure S7g), $E_F$ is close to the midgap and free electron concentration decreases with increasing number of electron traps becoming available to trap electrons. In this scenario, both electron and hole trap states are available to capture photogenerated carriers. At low laser power, a large fraction of electrons and holes get trapped first. Therefore, compared with $V_{gs}$ > 0 V, reduced photocurrent generation is observed as shown in Figure S7a and d. At higher laser power, the gradual filling of trap states results in more free photogenerated carriers in the channel. Finally, an increase of responsivity for higher laser power (Figure 5b and d) is observed. In total, we will get the nonmonotonic behavior shown in Figure 5b and d. According to the above analysis, we could expect that a lower charge carrier injection/extraction barrier would lead a higher probability of the existence of this nonmonotonic behavior. Here, because the inserting of graphene between metal and $MoTe_2$ ($WSe_2$) can significantly reduce SBH and thermionic field emission activation energies of carriers, we can easily observe this nonmonotonic behavior in both $MoTe_2$ and $WSe_2$ devices with G/M q-vdWC.

## References

[1] Q. S. Guo, A. Pospischil, M. Bhuiyan, H. Jiang, H. Tian, D. Farmer, B. C. Deng, C. Li, S. J. Han, H. Wang, Q. F. Xia, T. P. Ma, T. Mueller, F. N. Xia, Nano Lett. **2016**, 16, 4648.

[2] L. Yin, K. Xu, Y. Wen, Z. X. Wang, Y. Huang, F. Wang, T. A. Shifa, R. Q. Cheng, H. Ma, J. He, Appl. Phys. Lett. **2016**, 109, 213105.

[3] A. V. Penumatcha, R. B. Salazar, J. Appenzeller, Nat. Commun. **2015**, 6, 8948.
